# Survival outcomes after orbital exenteration for recurrent or previously treated orbital malignancies: impact of margin status and metastatic spread

**DOI:** 10.1007/s10006-026-01591-z

**Published:** 2026-07-03

**Authors:** M. Todaro, F. Perquoti, F. De Lorenzis, G. Gasparini, G. Saponaro, G. Barbera, G. Savino, M. G. Sammarco, M. M. Pagliara, G. Schinzari, E. Rossi, L. Tagliaferri, B. Fionda, F. Pastore, T. Tartaglione, L. Ausili Cefaro, X. Moix Gil, V. Santoro, A. Moro

**Affiliations:** 1 Maxillofacial Surgery Unit, Fondazione Policlinico Universitario A. Gemelli IRCCS Hospital, Rome, 00168 Italy; 2 Maxillofacial Surgery Unit, Head and Neck Department, University of Verona, Verona, 37134 Italy; 3 Ocular Oncology Unit, Fondazione Policlinico Universitario A. Gemelli IRCCS Hospital, Rome, 00168 Italy; 4 Ophthalmology Department, Università Cattolica del Sacro Cuore (UCSC), Rome, 00168 Italy; 5 Medical Oncology Unit, Fondazione Policlinico Universitario A. Gemelli IRCCS Hospital, Rome, 00168 Italy; 6 Radiation Oncology Unit, Department of Diagnostic Imaging, Radiation Oncology and Hematology, Fondazione Policlinico Universitario A. Gemelli IRCCS Hospital, Rome, 00168 Italy; 7 Radiology Department, Fondazione Policlinico Universitario A. Gemelli IRCCS Hospital, Rome, 00168 Italy; 8 Division of Maxillofacial Surgery, Department of Medicine, Surgery and Dentistry, ASST Santi Paolo e Carlo Hospital, University of Milan, Milan, Italy

**Keywords:** Orbital exenteration, Orbital malignancies, Prognostic factors, Eyelid carcinoma, Overall survival

## Abstract

**Background:**

Orbital exenteration is a radical procedure used in selected orbital malignancies, yet survival outcomes vary widely, particularly in recurrent or previously treated disease. We evaluated the association of pathological stage (nodal and/or distant metastasis status) and margin status with overall survival (OS) and disease-free survival (DFS) in patients undergoing exenteration for recurrent or previously treated orbital cancers.

**Methods:**

We retrospectively analyzed 39 patients who underwent orbital exenteration between 2017 and 2024. Patients were categorized as localized disease (pN0/pM0) or advanced disease with nodal and/or distant metastasis (pN + and/or pM+). Survival was estimated using Kaplan–Meier analysis and compared using the log-rank test. Median follow-up was 365 days. Margins were classified as R0 or R1.

**Results:**

Of the 39 patients, 24 (61.5%) had localized disease and 15 (38.5%) had advanced disease with nodal and/or distant metastasis; 6 patients (15.4%) had positive margins. 24-month OS was 78% in localized disease versus 33% in patients with nodal and/or distant metastasis (*p* = 0.011). Median OS was not reached in localized disease and was 13 months in patients with nodal and/or distant metastasis. 24-month DFS was 62.5% versus 33% (*p* = 0.017). R1 margins were associated with markedly worse outcomes: 24-month OS 0% vs. 75.8% for R0, and all R1 patients recurred within 12 months. Sensitivity analysis excluding < 6-month follow-up confirmed these findings.

**Conclusions:**

Nodal and/or distant metastatic status and positive surgical margins were associated with worse survival outcomes after orbital exenteration for recurrent or previously treated orbital malignancies. Most adverse events occurred within the first postoperative year, suggesting a clinically relevant early risk period. Given the histological heterogeneity of the cohort, these findings should be interpreted as prognostic patterns in a salvage orbital oncology population and require validation in larger disease-specific series.

## Introduction

**Fig. 1:**
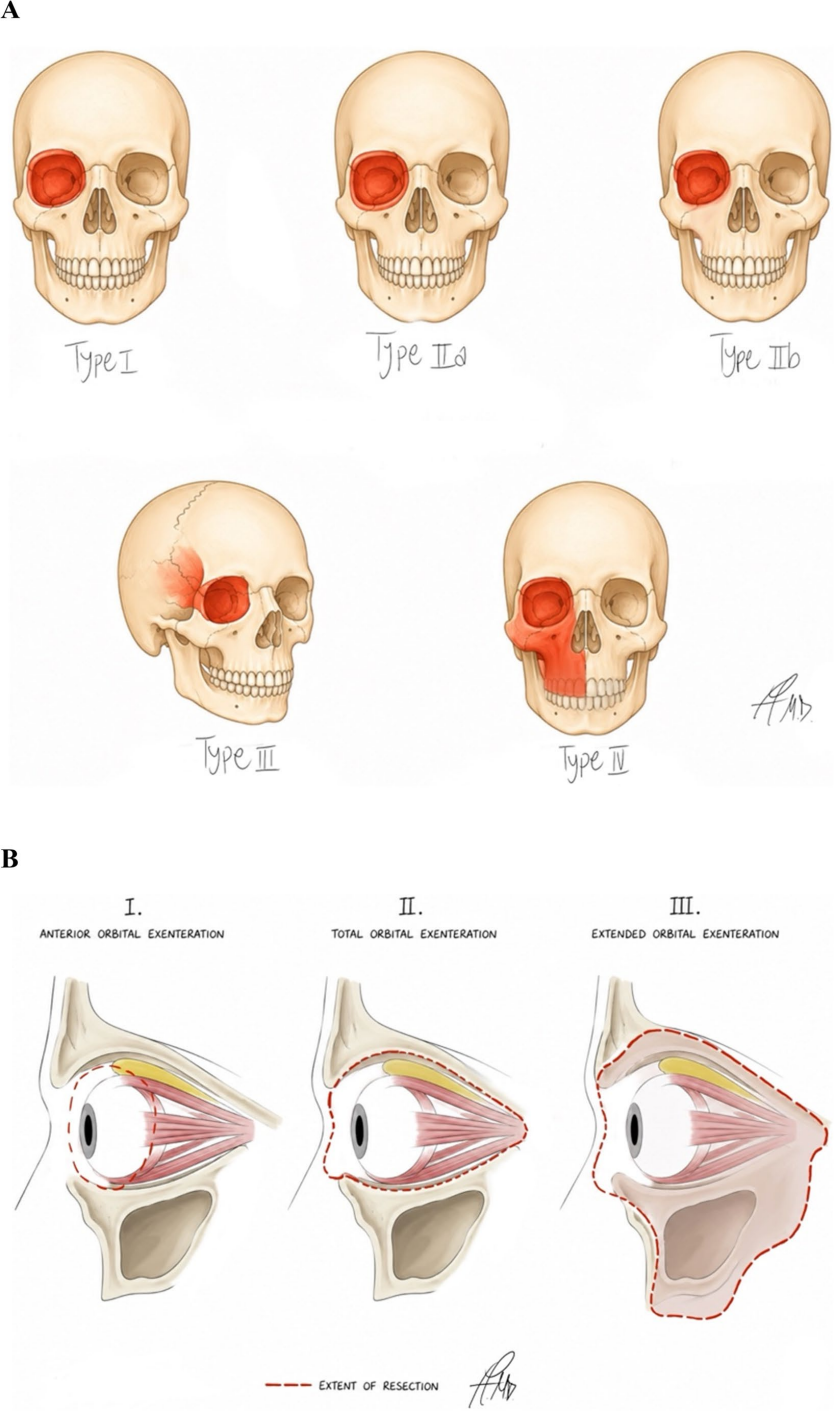
(**A**) Anatomical representations illustrating the Kesting classification: Type I (subtotal), removal of orbital contents with preservation of eyelids and conjunctiva; Type II (total), complete removal of all orbital tissues including the globe, extraocular muscles, and eyelids; Type III (extended), resection involving adjacent bone or facial structures in cases of extra-orbital invasion; and Type IV, combined with partial or total maxillectomy for extensive involvement of the orbital floor or paranasal sinuses. (**B**) Illustration representing I. Anterior orbital exenteration, II. Total orbital exenteration, and III. extended orbital exenteration

 Orbital exenteration is a radical surgical procedure involving complete removal of the orbital contents. First described by George Bartisch in 1583, it is now considered a high-impact intervention—both functionally and aesthetically—reserved for highly selected cases [1]. Over the centuries, the technique has undergone significant refinements: the modern concept of total exenteration was introduced by Golovine [2], and later systematized through the anatomically based classification proposed by Kesting et al. (Fig. 1) [3].

The annual incidence of orbital exenteration is approximately 0.1 per 100,000 inhabitants in Western populations, with higher rates among elderly individuals (mean age 65–75 years) and male predominance. More than 90% of exenterations are performed for malignant neoplasms—either primary or secondary orbital cancers—while benign or infectious indications have become uncommon [4].

Survival outcomes after orbital exenteration vary widely, ranging from 33% to 84%, and are influenced by tumor histology, pathological stage, surgical margin status, perineural or lymphovascular invasion, patient age, and therapeutic intent [5–7]. However, a persistent gap in the literature concerns patients who are heavily pre-treated, often with multiple prior surgeries or radiotherapy. In these individuals, prognosis is particularly uncertain due to altered anatomy, fibrosis, and the intrinsic aggressiveness of recurrent disease.

Recent reports have attempted to refine the indications for orbital exenteration and to clarify its prognostic implications in complex clinical scenarios [8, 9]. Nonetheless, the extent to which pathological staging (pTNM), margin status, and adjuvant therapies influence survival remains incompletely characterized—especially in pre-treated or recurrent cases. While advanced disease (defined by nodal involvement and/or distant metastases) is intuitively associated with poorer outcomes, only a limited number of studies have provided stratified survival data with adequate methodological detail [5, 10]. Similarly, the role of postoperative radiotherapy or chemotherapy in modifying long-term outcomes is still debated.

In this study, we aimed to provide a comprehensive survival analysis of 39 patients who underwent orbital exenteration for malignant orbital cancers, focusing on a population largely characterized by previous local treatments. By stratifying patients into localized (pN0/pM0; *n* = 24) and advanced (pN+/pM+; *n* = 15) disease groups, and by evaluating the impact of surgical margins and adjuvant therapies, we sought to identify key prognostic factors and delineate clinically relevant windows for optimal intervention and postoperative surveillance.

## Materials and methods

We conducted a retrospective cohort study including all consecutive patients who underwent orbital exenteration for histologically confirmed malignant orbital cancers at the Maxillofacial Surgery Unit, Fondazione Policlinico Universitario Agostino Gemelli IRCCS, Rome, Italy. All cases were discussed preoperatively within a multidisciplinary tumor board, and the indication for exenteration was based on tumor location, extent of disease, previous treatments, and expected impact on visual function and quality of life. In many patients, tumor size, recurrent bleeding, or failure of multiple prior conservative treatments performed elsewhere made exenteration the final feasible therapeutic option.

Between May 2017 and December 2024, 43 patients (31 males, 12 females) underwent orbital exenteration at our institution. Of these, 39 patients had complete pathological TNM (pTNM) staging and adequate follow-up data, and were therefore included in the survival analysis.

Patient-level variables collected included age, sex, tumour site, date and type of initial diagnosis, previous treatments, pTNM stage at surgery, histological subtype, surgical margins, recurrence type, adjuvant therapies, and follow-up duration.

The operated side was the right orbit in 23 cases and the left in 20 cases. Primary tumour sites were the conjunctiva (*n* = 17, 39.5%), eyelid (*n* = 15, 34.9%), lacrimal gland (*n* = 10, 23.3%), and other sites in 2.3%.

Histological confirmation was obtained in all patients through preoperative biopsy. Staging included contrast-enhanced MRI of the head and neck and contrast-enhanced whole-body CT.

In the original cohort of 43 patients, squamous cell carcinoma was the most frequent diagnosis (*n* = 22, 51.2%), followed by melanoma (*n* = 6, 14.0%). Basal cell carcinoma and adenocarcinoma accounted for 3 cases each (7.0%). The remaining nine cases (20.9%) included single occurrences of neuroendocrine carcinoma, poorly differentiated carcinoma, adenoid cystic carcinoma, sebaceous gland carcinoma, epithelial–myoepithelial carcinoma, sebaceous carcinoma, leiomyosarcoma, adenosquamous carcinoma, and porocarcinoma (Table 1). Among the 39 patients included in the survival analysis, SCC represented 56.4% of cases (*n* = 22/39).

**Table 1:** Histological distribution in the original cohort (*n*=43)

Category	Frequency	Percentage (%)
Squamous Cell Carcinoma	22	51.16%
Melanocytic Carcinoma	6	13.95%
Basal Cell Carcinoma	3	6.98%
Adenocarcinoma	3	6.98%
Other	9	20.93%

A considerable proportion of the cohort (27/43 patients) had undergone multiple prior treatments at other institutions. These included excisional procedures (*n* = 10), neoadjuvant chemotherapy (*n* = 15), and radiotherapy (*n* = 3), including brachytherapy. For 18 patients, orbital exenteration represented the last viable therapeutic option following failure of previous conservative approaches.

The most common presenting symptoms were ocular motility impairment, visual deterioration, and exophthalmos. The mean interval between the onset of symptoms prompting medical evaluation and exenteration was 1,153.5 days (~ 3 years) with a wide range of 14–6,779 days (~ 18 years; SD 1,357.4 days). Importantly, this interval began at the onset of symptoms, not at the time of diagnosis, and reflects multiple diagnostic and therapeutic steps, often carried out at external centers (*n* = 27 patients) before referral for definitive management.

Of the 39 patients included in the survival analysis, 24 were classified as having localized disease (pN0/pM0). Eleven had regional nodal metastasis without distant metastasis (pN + M0), and four had metastatic disease (pM+). For comparative analyses, patients were stratified into two groups based on pathological staging:

- Localized disease: pN0 and pM0, irrespective of primary tumor T category.
- Advanced disease: presence of nodal involvement (pN+) and/or distant metastasis (pM+), independent of T category.

Total exenteration involved removal of all orbital contents, including the eyelids. Extended exenteration was performed when there was invasion of bony orbital walls or adjacent maxillary or frontal structures, with en bloc resection as required. Following exenteration, reconstruction was performed using a temporalis muscle flap harvested via anterior retrograde approach [11]. In two patients the reconstruction was performed with microvascular flap (*n* = 1 Anterolateral Thigh Flap; *n* = 1 Latissimus Dorsi Flap), due to extended resection involving the temporalis flap.

Margin status was categorised histopathologically as:

- R0: no tumour cells at the resection margin.
- R1: microscopic residual disease.

Intraoperative serial margin sampling was performed in 26 of the 39 patients. This technique consisted of harvesting multiple elongated, thin tissue strips circumferentially from the tumour–healthy tissue interface, each submitted for frozen-section analysis. When tumour cells were identified at any sampled site, the resection was extended until intraoperative negative margins were achieved. This protocol was primarily applied in cases with invasion of periocular skin and adnexal structures or proximity to critical anatomical structures.

Previous systemic therapy was administered in 10 patients (25.6%), either with the intent to downstage the tumor or to delay surgery. Postoperative adjuvant therapy (radiotherapy and/or chemotherapy) was administered in 14 patients (35.9%) based on histological subtype, margin status, and pTNM stage: 8 in the localized group (33.3%), 4 in the N+ group (36.4%), and 2 of the 4 metastatic patients (50%), in whom treatment had a purely palliative intent.

**Fig. 2:**
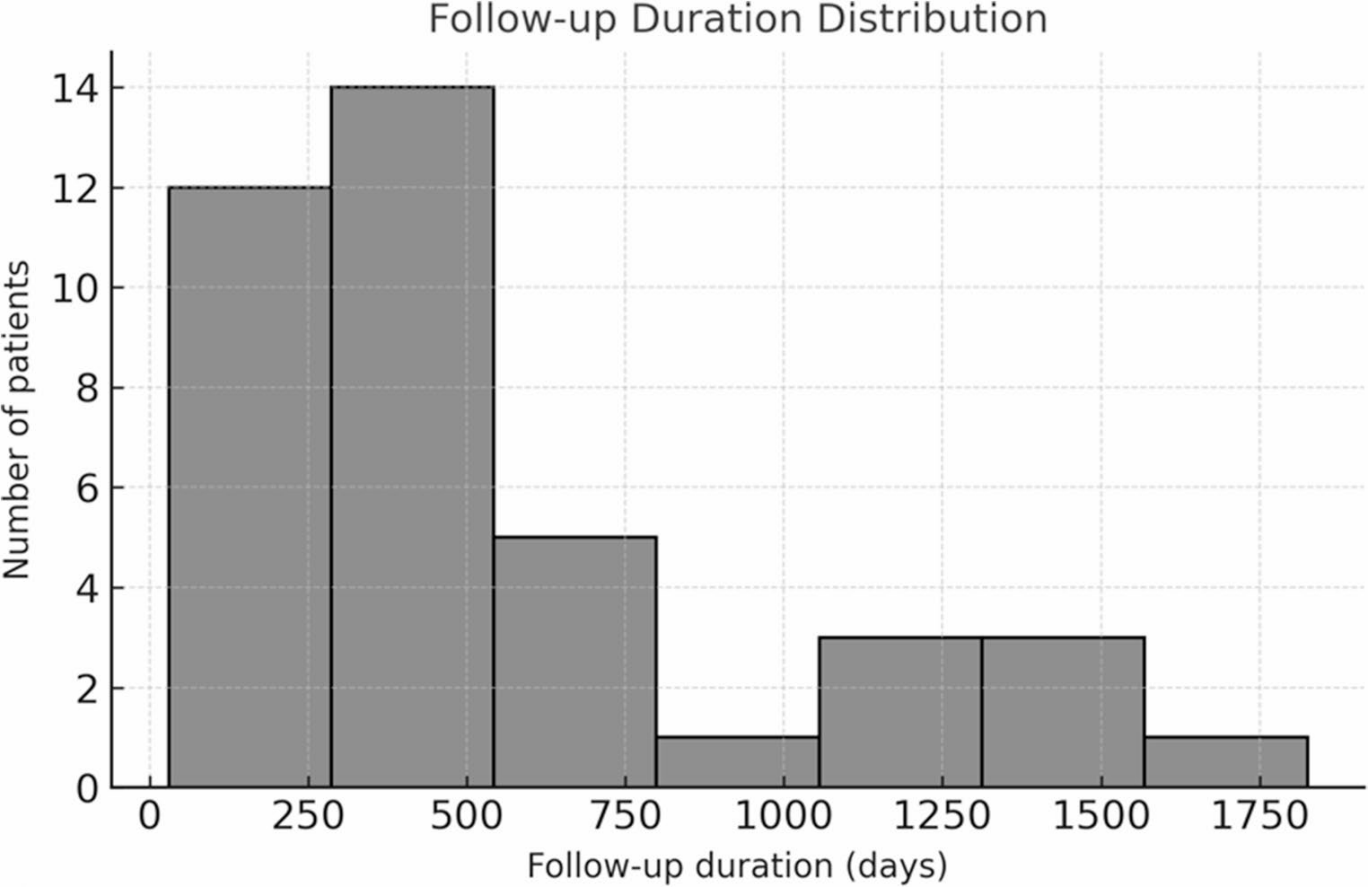
Distribution of follow-up duration (*n* = 39). Median follow-up = 12 months (range 30–1826)

Across the 39 patients included in the analysis, mean follow-up duration was 543 days (~ 18 months), with a median of 12 months (range: 1 month–5 years). Although some patients were followed for several years, the majority had observation times close to one year (Fig. 2).

Overall survival (OS) was defined as time from surgery to death from any cause. Disease-free survival (DFS) was defined as time from surgery to first recurrence or death. Recurrences occurred in 16 patients (*n* = 8 at the orbital level, *n* = 8 at lymph node level); one case was excluded from recurrence analysis due to incomplete follow-up (Table 2). Patients alive and free of disease at last contact were censored at that time.

**Table 2:** Clinical and pathological characteristics of patients undergoing orbital exenteration, including age, history, tumor site, disease group (Localized/Advanced), pTNM stage, OS Status, adjuvant therapy, follow-up time (days) and FUP Status

Patient	Age	Histology	Localization	Group	pTNM	OSStatus	Adiuvant	FUTime(Days)	FUPStatus
1	73	SCC	Eyelid	Localized	pT4a NO MO	0	RT	1096	NED
2	65	SCC	Conjunctiva	Localized	pT4a pNx pMx	0	.	1096	FOD
3	84	BCC	Eyelid	Localized	pT4a NO MO	0	.	730	FOD
4	84	UndifferentiatedCarcinoma	Eyelid	Localized	ypT3c NO MO	1	RT	365	DOD
5	45	SCC	Conjunctiva	Localized	pT4a NO cMO	0	.	730	NOD
6	72	SCC	Lacrimal Gland	Localized	pT1c N0 cMO	0	RT	1826	FOD
7	70	SCC	Eyelid	Localized	pT4b, Nx	1	RT	183	DOD
8	29	AdenocysticCarcinoma	Lacrimal Gland	Localized	cT2	0	RT	1461	NOD
9	70	Melanoma	Conjunctiva	Localized	pT2bNO	0	.	1461	NOD
10	88	SCC	Conjunctiva	Localized	pTis	0	.	1461	NOD
11	80	Sebaceous Carcinoma	Conjunctiva	Localized	pT4aNO Mx(non valutabile)	0	.	240	NOD
12	77	Melanoma	Conjunctiva	Localized	pT4eNO	1	.	30	DOD
13	55	Epithelial-MyoepithelialCarcinoma	Lacrimal Gland	Localized	pT2aNO	0	.	30	NOD
14	70	SCC	Eyelid	Localized	rcT4a	0	.	240	NOD
15	73	BCC	Eyelid	Localized	rcT4a	0	.	1096	NOD
16	73	SCC	Conjunctiva	Localized	pT3NOcMO	0	.	365	NOD
17	88	SCC	Eyelid	Localized	pT1aNOMO	0	.	365	EOD
18	99	Leiomyosarcoma	Eyelid	Localized	pT2NOMO	1	.	335	NOD
19	75	AdenosquamousCarcinoma	Lacrimal Gland	Localized	pT2aNOMO	0	CHT	335	EOD
20	80	SCC	Eyelid	Localized	pT3c pNXM0	0	.	183	NOD
21	61	NeuroendocrineCarcinoma	Ethmoid	Localized	pT4b	1	RT	183	DOD
22	80	SCC	Lacrimal Gland	Localized	pT1	0	.	730	FOD
23	81	SCC	Eyelid	Localized	pT4a	0	.	183	EOD
24	73	Melanoma	Eyelid	Localized	pT4b NO MO	1	RT/CHT	365	DOD
25	81	SCC	Conjunctiva	Advanced	pT3b N1 MO	1	.	730	DOD
26	72	Adenocarcinoma	Lacrimal Gland	Advanced	pT4c N1 cM0	1	RT	365	DOD
27	85	SCC	Eyelid	Advanced	cT4aN1b cMO	1	.	730	DOD
28	76	Porocarcinoma	Lacrimal Gland	Advanced	pT1b pN2bM0	0	.	365	NOD
29	76	SCC	Conjunctiva	Advanced	pT3N1MO	0	.	420	NOD
30	45	SCC	Conjunctiva	Advanced	pT4a pN1M0	0	RT	450	NOD
31	77	SCC	Conjunctiva	Advanced	pT4a pN2bM0	0	.	300	EOD
32	70	SCC	Conjunctiva	Advanced	pT4a pN1M0	0	.	300	NOD
33	74	SCC	Conjunctiva	Advanced	pT4a pN1M0	0	RT	300	NOD
34	69	SCC	Conjunctiva	Advanced	pT3pN1cM0	1	Immunotherapy	30	DOD
35	46	Adenocarcinoma	Lacrimal Gland	Advanced	pT2c pNx cM0	0	CHT	240	NOD
36	52	Adenocarcinoma	Lacrimal Gland	Advanced	pT4c N0 cM1	1	.	1096	DOD
37	76	SCC	Lacrimal Gland	Advanced	pT4b N0 pM1	1	.	365	DOD
38	52	Melanoma	Eyelid	Advanced	pT3cN0cM1	1	RT/CHT	30	DOD
39	59	SCC	Conjunctiva	Advanced	pT2N1pM1	1	.	122	DOD

Kaplan–Meier curves were generated for OS and DFS and compared using the log-rank test (two-sided, significance threshold *p* = 0.05). Median survival times, 24-month survival rates, and 95% confidence intervals (CIs) were calculated. An exploratory subgroup analysis examined OS and DFS in N + M0 (*n* = 11) and M+ (*n* = 4) patients; given the limited sample, no inferential tests were applied, and survival probabilities at 6 and 12 months were reported descriptively.

A sensitivity analysis excluding patients with < 6 months of follow-up was conducted to evaluate the robustness of findings. All analyses were performed using Python 3.10, employing Lifelines v0.27 for survival modelling and Matplotlib v3.8 for graphical representation.

Because of the histological heterogeneity of the cohort, an exploratory histology-specific descriptive analysis was performed for squamous cell carcinoma (SCC), which represented the largest subgroup. Other histological subgroups were not analyzed separately because of the limited number of cases. No inferential statistical comparisons were performed within histological subgroups.

### Management of nodal and distant metastatic disease

In patients presenting with regional nodal metastasis (pN+), nodal status was assessed preoperatively through imaging and confirmed histopathologically when neck dissection was performed. Management of the neck was individualized within a multidisciplinary tumor board, and included neck dissection and/or adjuvant radiotherapy according to tumor histology and overall staging.

In patients with distant metastasis (pM+), orbital exenteration was not performed with curative intent but for local disease control. Indications included control of bleeding, pain, ulceration, fungating tumor, or progressive orbital mass causing significant functional impairment. In these cases, surgery was undertaken despite known systemic dissemination, within a palliative or symptom-control framework.

## Results

The original cohort included 43 patients who underwent orbital exenteration for malignant orbital cancers. Four patients were excluded due to incomplete data, resulting in a final sample of 39 individuals. Among these, 24 were classified as having localized disease (pN0/pM0), while the remaining 15 had advanced disease, defined by the presence of nodal involvement (pN+), distant metastasis (pM+), or both.

Postoperative adjuvant therapy was administered to 14 patients. In the localized group, 8 patients (33.3%) received adjuvant treatment, whereas in the advanced group 4 N + M0 cases (36.4%) underwent adjuvant therapy and 2 metastatic patients (50%) received systemic treatment.

Intraoperative margin sampling was performed in 26 of the 39 patients (66.7%), in those cases where the tumor invaded periocular skin and adnexal structures. Frozen-section analysis identified microscopic tumor infiltration in 3 cases (11.5%), requiring enlargement of the resection margins. Final pathology revealed positive microscopic margins (R1) in six patients (15.4%). Five of these six individuals had undergone intraoperative serial margin sampling. Among these five, three showed positive frozen sections and underwent immediate resection extension; however, final deep permanent-section analysis still demonstrated microscopic residual disease. In the remaining two cases, frozen sections were negative intraoperatively, but final pathology revealed multifocal microscopic involvement in areas not captured by intraoperative sampling.

Overall survival analysis demonstrated a clear separation between the two groups. The two-year OS estimate was 78% for localized disease and 33% for advanced disease. Median OS was not reached for localized cases, indicating survival extending beyond the study window, whereas in advanced disease it was 13 months (95% CI: 9–18). This difference was statistically significant (log-rank *p* = 0.011). Margin status also strongly influenced prognosis: patients with R1 margins had a two-year OS of 0%, compared with 75.8% for those with R0 margins, and all six patients with positive margins died within two years of surgery. In all six R1 cases, further surgical clearance was considered technically unfeasible owing to the depth or multifocality of margin involvement. Consequently, adjuvant therapy represented the only viable postoperative approach for these patients.

**Fig. 3:**
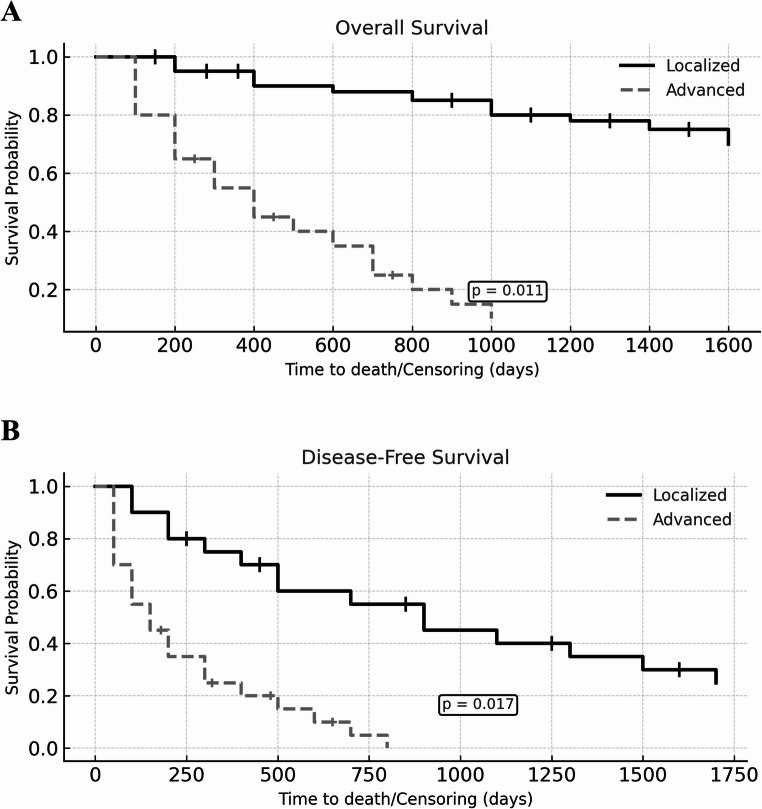
Kaplan–Meier survival curves for patients undergoing orbital exenteration for malignant orbital tumors. (**A**) Overall survival (OS) stratified by pathological stage (localized vs. advanced). (**B**) Disease-free survival (DFS) stratified by pathological stage. Localized disease (solid line) demonstrated significantly higher 24-month OS (78%) and DFS (62.5%) compared with advanced disease (33% and 33%, respectively).

Disease-free survival showed a similarly marked difference. At two years, DFS was 62.5% in the localized group and 33% in the advanced group. Median DFS was 18 months for localized disease and 8 months for advanced disease (95% CI: 5–13), with the difference again statistically significant (log-rank *p* = 0.017). As with OS, positive margins were associated with early recurrence, and all R1 patients experienced recurrence within the first postoperative year. Negative margins corresponded to a two-year DFS of 68% in localized disease, whereas advanced cases with R0 margins showed a two-year DFS of 46% (Fig. 3).

To assess the potential influence of short follow-up duration, a sensitivity analysis was performed excluding patients with less than six months of follow-up. Twenty-nine patients met this criterion, and the results remained consistent with the primary analysis. Localized disease continued to demonstrate a survival advantage, with a two-year mortality rate of 28% compared with 70% for advanced disease. DFS outcomes were unchanged, reinforcing the prognostic role of pTNM staging and margin status.

An exploratory descriptive analysis was undertaken to characterize survival patterns within patients presenting with nodal and/or distant metastasis. It included 11 patients with N + M0 disease and four with metastatic (M+) disease. Although limited by small numbers, the analysis highlighted distinct survival trajectories: median OS was approximately 13 months in the N + M0 subgroup and 7 months in the M+ subgroup. At one year, survival probabilities were around 45% and 20%, respectively. Median DFS was approximately 8 months for N + M0 disease and 6 months for metastatic disease, reflecting the tendency toward early recurrence or disease-related death in both categories. These findings align with expected clinical behavior in advanced-stage disease and, although exploratory, provide a useful descriptive framework for future studies.

Squamous cell carcinoma represented the largest histological subgroup in the final survival-analysis cohort (22/39, 56.4%). In this subgroup, the 24-month overall survival was 59.8%, and median overall survival was not reached. The 24-month disease-free survival was 48.7%, with a median disease-free survival of 730 days. Because of the limited sample size and the exploratory nature of this subgroup analysis, these findings should be considered descriptive and hypothesis-generating rather than inferential.

## Discussion

Orbital exenteration remains a disfiguring but sometimes indispensable procedure in the management of advanced orbital malignancies. Despite its profound functional and aesthetic consequences, it often represents the only potentially curative option when disease control cannot be achieved through more conservative treatments. Over time, management strategies have evolved toward increasingly individualized and multidisciplinary approaches integrating surgery with radiotherapy and systemic therapies [10, 12]. In this context, identifying prognostic factors that meaningfully influence survival is essential for guiding clinical decision-making.

In this retrospective single-center study, we evaluated survival outcomes in 39 patients undergoing orbital exenteration over an eight-year period. Pathological stage (pTNM) and surgical margin status emerged as the main determinants of prognosis. Patients with localized disease (pN0/pM0) showed substantially higher two-year OS (78%) and DFS (62.5%) compared with those presenting with nodal and/or distant metastasis (33% for both OS and DFS; *p* = 0.011 and *p* = 0.017), confirming the strong impact of disease extent at the time of surgery. While this association is well established in oncologic literature, our cohort is distinctive in being enriched with recurrent and previously treated tumors, a population in which prognostic stratification remains particularly challenging.

The histological heterogeneity of the cohort is an important consideration. Although mixed tumor types may complicate the interpretation of prognostic factors, this heterogeneity also reflects the clinical reality of orbital exenteration in tertiary referral practice. In our series, SCC was the most represented histology and was therefore analyzed descriptively as a disease-specific subgroup. The observed SCC survival pattern was broadly consistent with the overall cohort, suggesting that disease extent and margin status remain clinically relevant even within the largest histological category. However, these findings should be interpreted cautiously and should not be considered definitive histology-specific estimates.

A notable finding concerns the temporal pattern of disease progression. Most deaths and recurrences occurred within the first postoperative year, suggesting that this period deserves particular attention during follow-up. Similar early failure patterns have been described in periorbital and craniofacial malignancies, where rapid systemic progression significantly influences survival [13, 14].

Margin status had a profound impact on outcomes. Our findings are consistent with previous reports showing markedly reduced survival in patients with positive margins. Qedair et al. reported a significant difference between R0 and R1 resections (median OS 64 vs. 24 months) [5], while Ben et al. described margin positivity rates as high as 82% in complex exenteration cases [15]. Other multicenter studies have reported positive margins in 32–53% of patients [8, 16]. In our cohort, none of the six patients with R1 margins survived beyond two years despite adjuvant therapy, highlighting complete tumor clearance as the most relevant modifiable surgical factor.

Intraoperative serial margin sampling proved useful in guiding surgical decision-making. Frozen-section analysis detected microscopic tumor infiltration in three cases (11.5%), allowing immediate extension of resection margins. However, persistent R1 disease despite negative intraoperative findings in some patients underscores the limitations of frozen sections in the orbital region and the aggressive behavior of recurrent tumors. This technique should therefore be considered an adjunct rather than a guarantee of complete histopathological clearance [6].

Achieving clear margins in orbital surgery remains inherently challenging. Unlike eyelid or adnexal resections, where lateral margins are more easily defined, orbital resections often rely on the periosteum as a boundary, which may be insufficient in cases of microscopic tumor spread. These anatomical constraints contribute to variability in reported margin positivity rates and emphasize the need for individualized surgical strategies. In our series, re-excision was not feasible in any R1 case due to anatomical limitations or multifocal disease, making adjuvant therapy the only viable postoperative option.

The strong association observed between DFS and OS suggests that DFS may serve as an early surrogate endpoint in orbital malignancies. Early recurrence—particularly within the first postoperative year—was consistently associated with subsequent disease-specific mortality, in line with previous reports [17, 18].

When contextualized within existing literature, our survival outcomes and recurrence patterns are consistent with those reported in studies of eyelid carcinoma, lacrimal gland tumors, and mixed orbital malignancies [4, 14, 19]. The differences observed between localized and advanced disease align with recent multicenter analyses [9, 20], supporting the external validity of our findings.

Our exploratory analysis further highlighted a gradient of risk within advanced disease. Although limited by sample size, patients with nodal involvement without distant metastasis (N + M0) showed longer survival compared with those with metastatic disease (M+) [11]. This finding suggests that selected N + M0 patients may still benefit from aggressive local treatment, whereas systemic disease reflects a more advanced biological stage.

From a clinical perspective, early diagnosis and definitive treatment of localized disease remain critical. In advanced or recurrent cases, multidisciplinary management and careful follow-up are essential, particularly during the first postoperative year, when recurrence is most likely. In palliative settings, improvement of quality of life remains a key therapeutic goal.

## Limitations

This study is limited by its retrospective single-center design, limited sample size, and histological heterogeneity. The median follow-up was 12 months; therefore, 24-month OS and DFS estimates should be interpreted with caution, as the number of patients remaining at risk beyond the first postoperative year was limited. Although SCC represented the largest subgroup and was analyzed descriptively, the number of cases was insufficient for robust histology-specific survival comparisons or multivariable modeling. Therefore, the findings should be interpreted primarily as prognostic patterns within a salvage orbital oncology population rather than as definitive disease-specific estimates. In addition, reconstructive outcomes were not analyzed comparatively, as the study was designed to evaluate survival and oncological prognostic factors rather than flap-related endpoints. Larger multicenter studies focused on specific histological subgroups are needed to validate these observations.

## Conclusion

In this retrospective single-center cohort of recurrent or previously treated orbital malignancies, nodal and/or distant metastatic status and positive surgical margins were associated with worse overall and disease-free survival after orbital exenteration. Most adverse events occurred within the first postoperative year, suggesting a particularly vulnerable phase of follow-up. Although limited by sample size and histological heterogeneity, these findings may help refine postoperative risk stratification and support future disease-specific studies on the role of exenteration in salvage orbital oncology.

## Data Availability

Only to adjust form and grammatical sintax.
